# Data in the time of COVID-19: a general methodology to select and secure a NoSQL DBMS for medical data

**DOI:** 10.7717/peerj-cs.297

**Published:** 2020-09-10

**Authors:** Kamal A. ElDahshan, AbdAllah A. AlHabshy, Gaber E. Abutaleb

**Affiliations:** Mathematics Department, Faculty of Science, Al-Azhar University, Cairo, Egypt

**Keywords:** NoSQL databases, Database security, Key-value stores NoSQL systems, Document-based stores NoSQL systems, Column-based stores NoSQL systems, Graph stores NoSQL systems, Object Store NoSQL systems, COVID-19 patients’ data

## Abstract

**Background:**

As the COVID-19 crisis endures and the virus continues to spread globally, the need for collecting epidemiological data and patient information also grows exponentially. The race against the clock to find a cure and a vaccine to the disease means researchers require storage of increasingly large and diverse types of information; for doctors following patients, recording symptoms and reactions to treatments, the need for storage flexibility is only surpassed by the necessity of storage security. The volume, variety, and variability of COVID-19 patient data requires storage in NoSQL database management systems (DBMSs). But with a multitude of existing NoSQL DBMSs, there is no straightforward way for institutions to select the most appropriate. And more importantly, they suffer from security flaws that would render them inappropriate for the storage of confidential patient data.

**Motivation:**

This paper develops an innovative solution to remedy the aforementioned shortcomings. COVID-19 patients, as well as medical professionals, could be subjected to privacy-related risks, from abuse of their data to community bullying regarding their medical condition. Thus, in addition to being appropriately stored and analyzed, their data must imperatively be highly protected against misuse.

**Methods:**

This paper begins by explaining the five most popular categories of NoSQL databases. It also introduces the most popular NoSQL DBMS types related to each one of them. Moreover, this paper presents a comparative study of the different types of NoSQL DBMS, according to their strengths and weaknesses. This paper then introduces an algorithm that would assist hospitals, and medical and scientific authorities to choose the most appropriate type for storing patients’ information. This paper subsequently presents a set of functions, based on web services, offering a set of endpoints that include authentication, authorization, auditing, and encryption of information. These functions are powerful and effective, making them appropriate to store all the sensitive data related to patients.

**Results and Contributions:**

This paper presents an algorithm to select the most convenient NoSQL DBMS for COVID-19 patients, medical staff, and organizations data. In addition, the paper proposes innovative security solutions that eliminate the barriers to utilizing NoSQL DBMSs to store patients’ data. The proposed solutions resolve several security problems including authentication, authorization, auditing, and encryption. After implementing these security solutions, the use of NoSQL DBMSs will become a much more appropriate, safer, and affordable solution to storing and analyzing patients’ data, which would contribute greatly to the medical and research effort against COVID-19. This solution can be implemented for all types of NoSQL DBMSs; implementing it would result in highly securing patients’ data, and protecting them from any downsides related to data leakage.

## Introduction

The exponential increase in the number of patients infected with COVID-19, and the complex race towards finding treatment and prevention protocols, is a concern shared by all. Across the globe, researchers are collecting every shred of information related to the disease, and all data related to patients and their relations, which brings along the challenge of recording large swaths of information. The volume of data is expected to be immense—matched only by its diversity and variability. And as the number of patients increases, and alongside it, the breadth and diversity of data, medical organizations, researchers, and authorities will require storage systems offering greater flexibility, and which aren’t beholden to a strict set of options. As it stands, the list of COVID-19 symptoms remains ill-defined; this entails the need for a database management system (DBMS) that can handle unstructured data, namely, a NoSQL DBMS. But until now, NoSQL database systems still struggle with security issues; the lack of security of NoSQL DBMSs may make it an obstacle to its adoption as the preferred standard to store COVID-19 patients’ personal data.

This paper begins by explaining the five most popular categories of NoSQL databases, introducing the most popular NoSQL DBMS types under each. Features of NoSQL DBMS are briefly explained, with a strong emphasis on security. The paper addresses security problems such as authentication, authorization auditing, and encryption. Not only does the paper mention security vulnerabilities in each type, but it also lays out a comparative study of the different types of NoSQL DBMS, compares the strengths and weaknesses of each. This comparison is built on a set of criteria, namely Data Model, Owner Company, Open source(Y/N), Implementation Programming Language, Stable Release issue dates, Support Authentication, Support Authorization, Support Auditing, Support Data Encryption, and Query Types. The paper then moves to provide an algorithm that would help organizations choose the most appropriate type for storing COVID-19 patients’ information.

The purpose of this paper is to propose security solutions that resolve this weakness of NoSQL DBMSs, this eliminating the hurdle preventing medical organizations and other organizations storing patient information from using them. The proposed solution solves several security problems, including authentication, authorization, auditing, and encryption. The implementation of our security solutions will allow NoSQL DBMSs to become the more appropriate, safer, and affordable solution to store and analyze COVID-19 patients’ data.

## NoSQL Systems Categories and Their Security Issues

The NoSQL acronym was introduced in 2009, during an event on distributed databases (Lakshman & Malik, 2010). New technologies, such as Google’s Bigtable (Chang et al., 2008) were presented as being capable of managing large amounts of data. Research on NoSQL databases has blossomed since (Lourenço et al., 2015). In addition to the five major categories of NoSQL systems, some Multi-model NoSQL database systems, XML databases (Elmasri & Navathe, 2017), as well as other types of systems that have been available even before the term NoSQL came into use, grew in popularity and usage. This section thus discusses the five main categories of NoSQL systems, as well as the Multi-model database ones, with an emphasis on their security features, in the interest of patient data safety. This section will also address the most commonly-used NoSQL DBMSs in terms of security. NoSQL databases suffer from a variety of security issues (Sahafizadeh & Nematbakhsh, 2015). In NoSQL database systems, security mechanisms such as Authentication and data encryption are either weak or altogether inexistent.

First, we review possible attacks, and security mechanisms used to prevent them.

### Introduction to security issues in NoSQL DBMSs

#### NoSQL database management systems classification

**Figure 1:**
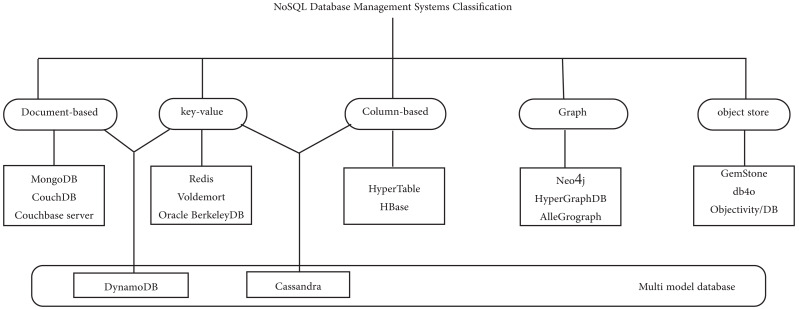
NoSQL Database Management Systems Classification.

NoSQL database management systems are classified into five categories, namely Document-based Stores, key-value stores, Column-based or wide column Stores, Graph-based Stores, and Object Stores. Multi-model database categories may be added as shown in Fig. 1.

#### NoSQL DBMSs comparison criteria

Database management systems will be analyzed according to their basic information, security mechanisms, and potential attacks.

• **Basic information includes:**
  – The owner and year of first appearance
  – The classification, specifying whether it is open source or proprietary
  – The DBMSs implementation programming language
  – Some characteristics of the database management system
  – The query language used in each database management system.
• **Security mechanisms**
  – **Authentication** is the process of verifying the entity identity (user or device) permission to use the resources (data, application, etc.) (Zahid, Masood & Shibli, 2014).
  – **Verification** is usually achieved through accessing control mechanisms, such as users’ passwords and certificates (Grolinger et al., 2013).
  – **Authorization** is the mechanism that ensures that only an authorized user is allowed to access system resources. Authorization is usually performed with the user’s permission (Grolinger et al., 2013).
  – **Auditing** is the process of keeping track and recording all actions performed by database users (Elmasri & Navathe, 2017).
  – **Data Encryption** refers to protecting and encrypting sensitive data throughout the communications network so it cannot be read by unauthorized users or attackers (Elmasri & Navathe, 2017; Grolinger et al., 2013). There are three levels of encryption solution; Data at rest, Client-to-server communication, and Server-to-server connections (Grolinger et al., 2013).
• **Attacks:** some attack types that DBMSs may endure include:
  – **Script Injection**, wherein a security vulnerability is used by an adversary to inject malicious code in pages or web forms to damage or retrieve data (Sullivan & Vincent, 2011; Jim, Nikhil & Michael, 2007).
  – **SQL Injection**, which may occur when an adversary injects a string input through the application to damage, change the database, retrieve the sensitive data, and run commands to deny service (Elmasri & Navathe, 2017).
  – **Denial of service** (DOS), in which an adversary floods the server with requests, thus preventing legitimate users from accessing the service, or the attacker may delete some data (Elmasri & Navathe, 2017).
  – **An inactive connection** may occur when an adversary opens the connection for a long time.

### Document-based NoSQL stores

Document-oriented, or document-based NoSQL systems store data as collections of similar documents. These types of systems are also known as document stores (Moniruzzaman & Hossain, 2013; Dindoliwala & Morena, 2017). Each document is similar to a row in relational databases, but is more flexible because it has no schema (is schema-free) and is specified as self-describing data (Elmasri & Navathe, 2017). The users or the processing applications have the schematic responsibility in document databases. This may result in some disadvantages, such as the lack of referential integrity and normalization. However, the lack of schema provides high flexibility to store a wide range of data, which makes it suitable for stores of big data. In document-based databases, a record, called a document, has its own internal structure, and can be stored as a value for every key (document ID). Documents are separated from one another, but are grouped as a collection of data, and the database ensures that each query receives the document version with the largest number of changes. Since this cannot guarantee full concurrency control, it is called eventual consistency. The consistency takes some time to achieve, which significantly speeds up the data processing at the expense of transactional security. In document-based databases, queries can be performed in parallel and, therefore, sped up with the map-reduce procedure (Meier & Kaufmann, 2019). Document-based databases are also fit for storing sparse data, or semi-structured data, but that would necessitate an extensive use of Nulls for data completeness purposes (Moniruzzaman & Hossain, 2013). Documents can be specified in various formats, such as XML (Extensible Markup Language), YAML (Yet Another Markup Language) (Kaur & Rani, 2013), or BSON (Binary JSON (JavaScript Object Notation) (Moniruzzaman & Hossain, 2013; Özsu & Valduriez, 2020). The popular language to specify documents in NoSQL systems is JSON (Han et al., 2011). Document-Based NoSQL Systems are suitable for managing Big Data collections of literal documents, like XML documents, email messages, text documents, online shopping, event logging, deep analytical processing, and content management. Examples of Document-Based NoSQL DBMSs (database management systems) include CouchDB (JSON), MongoDB (BSON) (Özsu & Valduriez, 2020), Terrastore, ThruDB, OrientDB, RavenDB, Citrusleaf, SisoDB, CloudKit, Perservere and Jackrabbit) (Tudorica & Bucur, 2011).

### Security issues in document-based NoSQL DBMSs

This subsection discusses the most popular types of document Stores NoSQL Database Management Systems.

#### MongoDB

• Introduction:
  MongoDB is an open-source document-oriented NoSQL DBMS. It is a schema-free, highly available, fault-tolerant and scalable NoSQL DBMS. MongoDB supports sharding by configuring shared clusters (Zahid, Masood & Shibli, 2014). It was developed by MongoDB Inc., first appearing in 2009; its stable release appeared in June 2018. MongoDB was written in C++, C, and JavaScript (Nayak, Poriya & Poojary, 2013; Özsu & Valduriez, 2020), and it consists of one or more collections of documents. A collection is analogous to a table, but has no pre-defined schema. The document, which is the data storing unit in MongoDB, has an ID and is equivalent to a record in the relational DB. Insert, delete, and update operations can be performed on a collection (Dindoliwala & Morena, 2017). Mongo Query Language is the query language used By MongoDB DBMS to manipulate certain documents from a database collection. Map-reduce and REST querying are supported by MongoDB (Chodorow, 2013).
• Security Analysis:
– Authentication: granting to users through the database itself or integration with an external mechanism like LDAP (Lightweight Directory Access Protocol). MongoDB grant Authentication to entities by using the SCRAM-SHA-1 IETF standard (Zugaj & Beichler, 2019). An authentication command is used to authenticate the users, whereas database-nodes are authenticated to the MongoDB cluster via key files. The default Authentication mechanism in MongoDB is SCRAM-SHA-1, which verifies the supplied user credentials with the name of the user, user’s password, and Authentication database. With LDAP, MongoDB can authenticate and authorize users directly via corporate LDAP infrastructure to enforce centralized access policies (Dindoliwala & Morena, 2017). MongoDB doesn’t support authentication, however, if running in shared mode (Sahafizadeh & Nematbakhsh, 2015; Noiumkar & Tawatchai, 2014). When the authentication in MongoDB is based on the key or the password, it’s called a pre-shared secret. This password has been hashed with the MD5 algorithm before being stored in the key file. Hackers can crack the pre-shared secret’s hashed value by cracking the MD5 (Dindoliwala & Morena, 2017), because the MD5 algorithm is not secured (Sahafizadeh & Nematbakhsh, 2015). MongoDB uses SSL with X.509 certificates to secure communication between the user and the MongoDB cluster and intra-cluster authentication (Zahid, Masood & Shibli, 2014).
– Authorization: Administrators in MongoDB can determine the permissions for users or applications. They can also decide what data can be seen by users or applications when performing a database query. The authorization mechanism in MongoDB contains LDAP authorization, roles, and Field-Level Security with Read-Only Views. In MongoDB privileges are assigned to roles, then roles are assigned to users. MongoDB involves built-in roles that are used by administrators to control access to the MongoDB system. A privilege consists of a specified resource and actions permitted on this resource. A resource is a collection, set of collections, databases, or a cluster. MongoDB supports a simple role-based authentication system that allows administrators to decide who has access to the database and which level of access they have. Furthermore, MongoDB supports authorization through LDAP (Dindoliwala & Morena, 2017).
– Auditing: Auditing framework stores actions such as operations that have been performed by the users in the database, and authentication and authorization activities along with write and read operations on the database. Administrators can extract and filter audit trails for any operation with MongoDB. For example, it is possible to audit and log the identities of users who accessed specific documents and the changes they made to the database during their session. Administrators can configure MongoDB to log all the actions or specific actions. MongoDB Enterprise Advanced (Dindoliwala & Morena, 2017) also supports the role-based auditing. With MongoDB, it is possible to report the DB activities as well.
– Data Encryption: in MongoDB, data can be encrypted over the network, in backups, and the rest in permanent storage. MongoDB Enterprise Advanced supports FIPS 140-2 encryption when it run in FIPS Mode (Dindoliwala & Morena, 2017). In MongoDB, the data files are not encrypted automatically, so all data is stored as plaintext. This means that any malicious user with access to the file system can extract information from the files. MongoDB uses RESTful to manage its server via the HTTP protocol. However, there is no data encryption for these ports, which means that hackers can display, capture and get access to the data since no data encryption was performed while the data was being sent back and forth between the client and server (Noiumkar & Tawatchai, 2014).
– Script Injection: Using JavaScript, hackers can attack MongoDB. Examples about the attacks that occurred on MongoDB can be found in this reference (Jim, Nikhil & Michael, 2007). NoSQL databases are exposed to injection attacks like SQL databases. Son presented four examples of GitHub site for injection vulnerabilities in PHP MongoDB-based applications (Son, McKinley & Shmatikov, 2013).

#### CouchDB

• Introduction:
  CouchDB is an open-source NoSQL DBMS. It was developed using Erlang (George, 2013). It became an Apache project in 2008. It was released in 2005 and the stable release appeared in November 2017. CouchDB runs on Hadoop Distributed File Systems (HDFS). CouchDB is document-based NoSQL DBMS, fault-tolerant, highly scalable, available, and flexible (Sahafizadeh & Nematbakhsh, 2015). It supports document redistribution across nodes by cluster configuration for large performance improvements. A cluster uses the phenomenon of incremental replication by periodically copying any changes in a document on a single node to other nodes. The incremental replication results in inconsistency and data redundancy in the cluster. CouchDB uses the JavaScript, JSON, and SQL++ as its query language (Özsu & Valduriez, 2020). Map-reduce and REST querying are supported by CouchDB as well (Noiumkar & Tawatchai, 2014).
• Security Analysis:
– Authentication: CouchDB uses a process called CRUD to perform authentication (Noiumkar & Tawatchai, 2014). The authentication in CouchDB is based on both cookies and passwords. It uses the PBKDF2 hash algorithm to encrypt the password and send it over the network using SSL protocol (Sahafizadeh & Nematbakhsh, 2015; Zahid, Masood & Shibli, 2014).
– Authorization: There are three levels of users within the CouchDB; database admin, server admin, and database member (Grolinger et al., 2013). Authorization is implemented only at the database level where only a single role in access control is supported (Zahid, Masood & Shibli, 2014).
– Auditing: The level of auditing in CouchDB is moderate (logging of all changes to user-profiles and the level of database). Auditing is provided to log events and views in log files. However, CouchDB does not support automatic backups of database logs, replicas, and automatic logging; the configuration of logs is thus the responsibility of the database administrators (Zahid, Masood & Shibli, 2014).
– Data Encryption: CouchDB does not have automatic data encryption, so the data files are at risk of being accessed and read directly (Sahafizadeh & Nematbakhsh, 2015; Noiumkar & Tawatchai, 2014). Data Encryption is supported in Client/Server via SSL. Also, data encryption could be supported using HTTPS connections in Server/Server (Grolinger et al., 2013).
– Denial of service attack: Websites such as securityfocus.com display vulnerabilities that exist only on Apache CouchDB 1.5.0 (SecurityFocus, 2020). These vulnerabilities are used by hackers to crash it. So CouchDB is potentially vulnerable to Denial of service attacks (Sahafizadeh & Nematbakhsh, 2015).
– Script Injection: CouchDB uses JSON to manipulate the data, making it vulnerable to Script Injection. In other words, hackers can launch JSON Injection to attack CouchDB (Sahafizadeh & Nematbakhsh, 2015; Noiumkar & Tawatchai, 2014).

#### Couchbase server

• Introduction:
  Couchbase Server is an open-source, document-oriented NoSQL DBMS. Couchbase Server was developed by Couchbase Inc., initially released in August 2010 with the stable release launched in February 2018. Couchbase Server was developed using Erlang, C, and C++ (George, 2013). Various data centers contain all the cluster servers. The documents are stored in vbuckets, which are special data containers uniformly distributed across the cluster. More nodes can be added and removed because Couchbase cluster scales are completely horizontal (Zahid, Masood & Shibli, 2014). Map-reduce and REST querying is supported by the Couchbase server (Grolinger et al., 2013). Couchbase also supports SQL++, an SQL like language which has been designed to become a unified query language for NoSQL system.
• Security analysis:
– Authentication: SASL (Simple Authentication and Security Layer) is supported by name and password on Couchbase Server HTTP. Basic authentication is used in Couchbase management REST (Representational State Transfer) API (Application Programming Interface) (Grolinger et al., 2013). External Authentication is also supported (Zahid, Masood & Shibli, 2014).
– Authorization: Couchbase Server does not support authorization (Grolinger et al., 2013).
– Auditing: Couchbase Server does not support auditing (Grolinger et al., 2013).
– Data Encryption: Couchbase Server does not support data Encryption in Data at rest, Client/Server, and Server/Server (Grolinger et al., 2013).

### Key-value NoSQL stores

These are the simplest NoSQL databases. Key-value stores focus on high availability, performance, and scalability, by storing data in a distributed storage system (Abed, 2020). The data model used in key-value stores is simple, and in most such systems, there is no query language, but rather a set of operations that can be used by the application programmers. The key is a unique identifier associated with a value, and is used to rapidly locate it (Kaur & Rani, 2013). The value may have different formats for different key-value storage systems. In some cases, the value is just an array or a string of bytes. Key-value stores are suitable for efficient reading and writing of extensive amounts of data. Some disadvantages of this model include the lack of some traditional capabilities such as atomicity and consistency, and as the data volume increases, retaining more values as keys may become more difficult (Dindoliwala & Morena, 2017). The application using the key-value store has to interpret the structure of the data value. Different key-value stores can thus store structured, unstructured, or semi-structured data items (Elmasri & Navathe, 2017). NoSQL Key-Value stores are needed for application tasks such as managing user profiles, sessions, or retrieving product names (Moniruzzaman & Hossain, 2013). Examples of this type of NoSQL DBMSs include Redis, Voldemort (LinkedIn), Riak, and BerkeleyDB (Özsu & Valduriez, 2020).

### Security issues in key-value stores NoSQL DBMSs

This subsection discusses the most popular types of Key-Value Stores NoSQL Database Management Systems.

#### Redis

• Introduction:
  Redis is an Open source NoSQL DBMS (Grolinger et al., 2013), developed using C and C++ (Meier & Kaufmann, 2019) by Salvatore Sanfilippo. Redis was initially released in May 2009 and the stable release appeared in June 2018. It is a Key-value memory database, So, Redis data will be loaded into memory (Dayan, Manos & Stratos, 2018) when it runs and all operations are run in memory. Redis periodically saves data asynchronously to the hard disk. It achieves high-performance thanks to its use of pure memory. Redis can handle more than 100,000 write or read operations per second. The maximum limit of value is 1GB. However, Redis cannot be used as big data storage, and scalability is poor because the capacity of the Database is limited by the physical memory. As such, Redis is suitable for providing high-performance computing for a small amount of data (Han et al., 2011). SQL-like and Map-reduce querying is not supported in Redis but REST querying is supported by third-party APIs (Grolinger et al., 2013).
• Security analysis:
  – Authentication: Redis provides a password-based authentication to its clients. Passwords are set by system administrators and stored in plaintext format. Redis does not provide default authentication and listens on all IP addresses on port 6739 (Han et al., 2011).
  – Authorization: Redis does not support any kind of access control (Han et al., 2011) i.e., it does not support authorization (Paterson et al., 2006).
  – Auditing: Redis does not provide support for auditing (Tudorica & Bucur, 2011; Paterson et al., 2006).
  – Data Encryption: Redis does not provide support for Data encryption. It stores data in plaintext (Paterson et al., 2006). As such, hackers—or anyone who can access the Redis server - will be able to read all the data in the database. No data encryption is performed in the communication between client and server, and between other servers, neither at the same cluster nor different clusters (Nayak, Poriya & Poojary, 2013). Redis does not have data-at-rest encryption (Zugaj & Beichler, 2019).
  – Denial of service attack: Denial of service attacks on Redis was not reported (Nayak, Poriya & Poojary, 2013).
  – Script Injection: Redis does not have a concept of string escaping, so injection becomes impossible (Nayak, Poriya & Poojary, 2013; Paterson et al., 2006).

#### Voldemort

• Introduction:
  Voldemort is an Open source key-value NoSQL DBMS, developed by LinkedIn (Dayan, Manos & Stratos, 2018). Its initial release was in 2009 and the stable release appeared in July 2017 (Grolinger et al., 2013). Voldemort was developed using Java (Deka, 2013). Three simple operations are included in Voldemort; reading, writing, and deletion. All of them are executed using a key (Chen, Shiwen & Yunhao, 2014). There is no SQL-like querying language in Voldemort but Map-reduce querying is supported (Grolinger et al., 2013).
• Security Analysis:
  – Authentication: There is no authentication mechanism in Voldemort.
  – Authorization: There is no authorization mechanism in Voldemort.
  – Auditing: There is no auditing in Voldemort.
  – Data Encryption: No encryption, neither between the client and the server nor among the servers (Grolinger et al., 2013).

#### Oracle Berkeley DB (BDB)

• Introduction:
  Oracle Berkeley DB is a closed source key-value NoSQL DBMS. Oracle Berkeley DB was developed by Sleepycat Software. Oracle Berkeley DB initial release appeared in 1994 and the stable release was in June 2018. It is a high-performance data management services to applications. It uses a simple function for data access and management (Sullivan & Vincent, 2011). SQLite and REST querying is supported by Oracle Berkeley DB, but Map-reduce querying is not (Grolinger et al., 2013). In terms of implementation of Oracle Berkeley DB, was implemented in C, Oracle Berkeley XML was implemented in C++ and Oracle Berkeley JE was implemented in Java.
• Security Analysis:
  – Authentication: There is no authentication mechanism in Berkeley DB.
  – Authorization: There is no authorization mechanism in Berkeley DB.
  – Auditing: There is no auditing in Berkeley DB.
  – Data Encryption: Berkeley DB supports data at rest encryption. No encryption between Servers (Grolinger et al., 2013).

### NoSQL graph stores

A graph database is a database that uses graph structures for semantic queries with nodes, edges, and properties to represent and store data. A key concept of this system is the graph, which relates data items in the store. Both edges and nodes can be labeled to indicate the types of vertices and associations they represent, and it is generally possible to store data associated with both individual nodes and individual edges (Elmasri & Navathe, 2017). In the Graph database model, the database is represented as a network structure containing edges between nodes to illustrate the relationships among nodes. Nodes may also contain properties that describe the real data contained within each object. Edges may also have properties. An edge (or a relationship) connects two nodes and it may be directed. When directions are added, relationships between nodes are identified by the names of the nodes, and can be traversed in both directions (Kaur & Rani, 2013). The graph uses the Index-Free adjacency technique, in which each node contains a pointer pointing to the adjacent node. For every node in the Graph database, the system can access its direct neighbor, without the need to consider all edges (Abed, 2020). This is known as the index-free adjacency property; and it is the key feature of the graph databases (Meier & Kaufmann, 2019). Graph databases are efficient schema-less DBs, which makes them suitable for semi-structured data storage. The queries in Graph databases are expressed as traversals; which make them much faster than relational databases (Nayak, Poriya & Poojary, 2013). In general, graph databases are appropriate when the main interest is the relationships between data (e.g., generating recommendations, social networks, conducting forensic investigations, network search, and fraud detection (Moniruzzaman & Hossain, 2013). Examples of Graph Databases include Neo4j, Sones GraphDB, InfiniteGraph, InfoGrid, and AllegroGraph (Khasawneh, AL-Sahlee & Safia, 2020).

### Security issues in graph NoSQL DBMSs

This subsection discusses the most popular types of graph Stores NoSQL Database Management Systems.

#### Neo4j

• Introduction:
  Neo4j is an open-source graph NoSQL DBMS (Paterson et al., 2006), developed by Neo Technology. It was initially released in 2007 and the stable release appeared in April 2018. It was developed using Java. The advantages of Neo4j include providing object-oriented, flexible network structure, reliability, highly available, and scalability. Neo4j uses the graph data model which consists of nodes and edges and the relationships between them. Neo4j uses CYPHER query language. REST querying is supported by Neo4j but Map-reduce is not supported (Grolinger et al., 2013). Neo4j is used extensively in software that has complex relationships such as social networking and recommendation engines (Moniruzzaman & Hossain, 2013).
• Security Analysis:
  – Authentication: Neo4j does not support Authentication (Grolinger et al., 2013).
  – Authorization: Neo4j does not support authorization (Grolinger et al., 2013; Paterson et al., 2006)
  – Auditing: Neo4j does not support auditing (Grolinger et al., 2013; Paterson et al., 2006).
  – Data Encryption: Neo4j does not support data at rest encryption, nor does it support data encryption between servers (Grolinger et al., 2013); however it uses SSL-based communication protocol between the client and the server (Paterson et al., 2006).

#### HyperGraphDB

• Introduction:

HyperGraphDB is an Open source NoSQL DBMS that implements powerful knowledge management formalism known as directed hypergraphs (Grolinger et al., 2013). HyperGraphDB is developed by Kobrix Software, Inc. The stable release of HyperGraphDB appeared in May 2017. Hyper-GraphDB was developed using Java. Higher-order Relations are naturally represented by the HyperGraphDB model. The HyperGraphDB model is very useful in areas related to data modeling such as artificial intelligence and knowledge of representation and bioinformatics. This data model is designed for AI, Knowledge representation and the semantic Web (Kobrix Software Inc, 2020; Angles, 2012). HyperGraphDB provides SQL- like query language. REST querying is supported by HyperGraphDB but Map-reduce is not (Grolinger et al., 2013).

• Security Analysis:
  –Authentication: HyperGraphDB does not support authentication.
  –Authorization: HyperGraphDB does not support authorization.
  –Auditing: HyperGraphDB does not support auditing.
  – Data Encryption: HyperGraphDB does not support data encryption (Grolinger et al., 2013).

#### AllegroGraph

• Introduction:
  AllegroGraph is a closed-source graph NoSQL DBMS, developed by Franz Inc. The stable release appeared in October 2017 (Khasawneh, AL-Sahlee & Safia, 2020; Grolinger et al., 2013). It was developed using Common Lisp (Franz Inc, 2020). It offers high-performance and is mainly used for developing semantic web applications. It can store data and meta-data as RDF (Resource Description Framework) triples. AllegroGraph maintains high performance when accessing billions of quads due to efficient memory utilization along with disk-based storage (Arora & Rinkle, 2013; Franz Inc, 2020). SPARQL and Prolog are the query languages used by AllegroGraph. REST querying is supported by AllegroGraph but Map-reduce is not (Grolinger et al., 2013).
• Security Analysis:
– Authentication: Authentication is supported by AllegroGraph.
– Authorization: Authorization is supported by AllegroGraph such as read, write, and delete.
– Auditing: AllegroGraph can be used to record specific changes in the audit log.
– Data Encryption: AllegroGraph does not support data at rest encryption. But it uses an HTTPS-based communication protocol between the client and the server (Grolinger et al., 2013).

### Column-based or wide column NoSQL stores

By providing an additional structure, Column-based databases utilize the key-value concept. In certain uses, it has been proven that to optimize the read operations, it is much better to store the data into the relational datasets per column, not per row. This is due to the occasional need for all columns in a single row at once; however, there are groups of columns that are often read together (Meier & Kaufmann, 2019). Column-based data stores are designed to address the huge number of columns and frequent changes in the schema (Moniruzzaman & Hossain, 2013). Wide column stores have tables that contain columns. A column is specified by a combination of a Column Family and a Column Qualifier; each column family has a name that should be declared when the table is created, and cannot be changed subsequently. After loading data into a table, each column family is associated with column qualifiers. Column qualifiers are not declared when the table is created, but can be dynamically created and inserted into the table. Read and write are done using columns rather than rows. The reason for structuring the data in groups of columns—column families—as storage units is that it is a sound way to optimize the data access. Column-based databases adopt this model; they store the data not in relational datasets but rather in enhanced and structured multi-dimensional keyspaces (Meier & Kaufmann, 2019). Wide-column stores have great features such as high performance and high scalability (Elmasri & Navathe, 2017). These types of DBMSs are suitable for data processing, distributed data storage, content management, event logging, and categorizing for analytics (Moniruzzaman & Hossain, 2013). The most common open-source column-oriented databases are HBase, Hypertable. HBase are derivatives of Bigtable (Kaur & Rani, 2013).

### Security issues in column-based NoSQL DBMSs

This subsection discusses the most popular types of column-based Stores NoSQL Database Management Systems.

#### Hypertable

• Introduction:
  Hypertable is a column-oriented NoSQL DBMS. It was developed by Zvents before 2008 and the stable release appeared in March 2016. Hypertable was developed using C++ (Deka, 2013). It is a high-performance open-source DBMS, and can run on Hadoop Distributed File Systems (HDFS). It is modeled after Google’s Bigtable DBMS. Like Bigtable, Hypertable stores data using the column-oriented methodology (Khasawneh, AL-Sahlee & Safia, 2020). There is no SQL-like query language in Hypertable.
• Security Analysis:
– Authentication: Hypertable does not support Authentication (Nayak, Poriya & Poojary, 2013; Paterson et al., 2006). Moreover, it does not support Authentication in communication between the client and the server or among its servers (Nayak, Poriya & Poojary, 2013).
– Data Encryption: Hypertable does not support encryption for its data files (Nayak, Poriya & Poojary, 2013; Paterson et al., 2006). Hypertable does not support encryption in communication between the client and server or between its servers (Nayak, Poriya & Poojary, 2013).
– Denial of service attack: There is no information reported about denial of service attacks on Hypertable.
– Script Injection: Though Hypertable has an HQL (Hypertable Query Language), which is similar to the SQL, it is not vulnerable to script injection (Nayak, Poriya & Poojary, 2013).

#### HBase

• Introduction:
  HBase is an open-source column-oriented NoSQL DBMS. HBase was developed by the Apache Software Foundation (2020). Its initial release was in February 2015 and the stable release was in April 2018. It was developed using Java (Meier & Kaufmann, 2019). Features include automatic distributability and scalability. HBase is based on the concept of Google’s Bigtable and implemented in Java (Tudorica & Bucur, 2011; Paterson et al., 2006). There is no SQL-like query language in HBase, but Map reduce and REST querying are supported (Grolinger et al., 2013).
• Security Analysis:
– Authentication: HBase supports token-based Authentication, for MapReduce tasks and user Authentication is done by using SASL (Simple Authentication and Security Layer with Kerberos Tudorica & Bucur, 2011; Paterson et al., 2006).
– Authorization: HBase supports authorization by access control list. Permissions include create, read, write and admin (Grolinger et al., 2013; Paterson et al., 2006).
– Auditing: Auditing is supported by HBase (Han et al., 2011).
– Data Encryption: HBase does not support the encryption of data at rest (Tudorica & Bucur, 2011; Paterson et al., 2006), but HBase supports the encryption in communication between client and server (Grolinger et al., 2013).
– Denial of service attack: No report for denial of service attack.
– Script Injection: No report for script injection (Paterson et al., 2006).

### Object store NoSQL stores

An object-oriented database stores the information or data as objects according to the object-oriented paradigm (Nayak, Poriya & Poojary, 2013). Object-oriented stores can be thought of as a combination of object-oriented programming (OOP) methodology and database principles. Data encapsulation, polymorphism, inheritance, and all other features of OOP are offered by other object-based databases. The classes, attributes, and objects in the Object-oriented stores are comparable to tables, columns, and tuples in a table in RDBMS respectively. Each object has an identifier that uniquely represents it. Because the object can easily be retrieved using pointers, data access becomes much faster in object-oriented databases. Harnessing the Object-based databases can ease the agility of modern software development processes (Nayak, Poriya & Poojary, 2013). These types of data stores are not relational databases and are not queried using SQL (Dindoliwala & Morena, 2017). Object Stores are the most convenient category for storing and retrieving binary large objects such as files, images, videos, and audio files. They are ideal for applications that require complex relationships between objects and changing object structures, or if the application defines members that are grouped in collections. Common uses for the object-oriented databases are applications of scientific research, telecommunication, computer-aided drafting, etc. The disadvantage of object-oriented databases is the limited programming languages they are bounded to (Nayak, Poriya & Poojary, 2013). Examples of Object Store NoSQL DBMSs include Gemstone, db4o, and objective/DB (Dindoliwala & Morena, 2017).

### Security issues in object store NoSQL DBMSs

This subsection discusses the most popular types of object Stores NoSQL Database Management Systems.

#### Gemstone

• Introduction:
  Gemstone is an open-source an Object NoSQL DBMS. It was developed by GemTalk Systems. Its first appearance was in 1986 and the stable release is 64 Bit 3.3.7. Gemstone is Proprietary commercial software. Multiuser environments are supported by Gemstone; each user can have one or more sessions, and multiple user sessions can be active at the same time. Gemstone provides security at several levels from login authorization to object access (Dindoliwala & Morena, 2017). There is no general query language like SQL in Gemstone.
• Security Analysis:
– Authentication: In Gemstone, each user is identified by a unique user ID and a password. The user is represented by an instance of the class User Profile. The User Profile contains a user ID, password, default authorization information, and the user’s group. Only users who have a User Profile can logon to the system.
– Authorization: Authorization exists within Gemstone and controls individual object access.
– Auditing: The DB administrator can also configure the Gemstone system to monitor failures to login.
– Data Encryption: Gemstone does not support Data Encryption (Dindoliwala & Morena, 2017).

#### db4o

• Introduction:
  db4o is an open-source object NoSQL DBMS. It was developed by Action and the stable release 8.0. db4o was developed using Java and C# (Noiumkar & Tawatchai, 2014). db4o-SQL is an interface to allow SQL queries into a db4o database.
• Security Analysis:
– Authentication: db4o provides only an internal authentication mechanism.
– Authorization: db4o offers file-level authorization.
– Auditing: db4o lacks auditing capabilities.
– Data Encryption: db4o uses the eXtended Tiny Encryption Algorithm (Dindoliwala & Morena, 2017) for data encryption.

#### Objectivity/DB

• Introduction:
  Objectivity/DB is an object NoSQL DBMS. Objectivity/DB is proprietary to Objectivity Inc. and first commercialized in 1990. Objectivity/DB was developed using Java, C#, C++, and Python. Since the Objectivity/DB schema is created from internal class definitions from the programming language, it is easier to maintain changes to the schema (Dindoliwala & Morena, 2017). Objectivity/DB provides OQL (Object Query Language which is like SQL querying language
• Security Analysis:
  – Authentication: For Authentication Objectivity/DB has two mechanisms; the first (by default) is Kerberos; the second is by using Advanced Multithreaded Server (AMSprotocol).
  – Authorization: Relies on the operating system and file systems for access control.
  – Auditing: No auditing capabilities have been mentioned in Objectivity/DB.
  – Data Encryption: Objectivity/DB doesn’t provide any inbuilt encryption (Dindoliwala & Morena, 2017).

### Multi-model NoSQL database Stores

This term refers to at least two types of NoSQL categories integrated into one system. In some of the NoSQL systems, a combination of more than one NoSQL system is used. This integration ensures that all the features in each category are combined. For example, Cassandra is a NoSQL DBMS used on Facebook. It combines column stores and key-value stores (Nayak, Poriya & Poojary, 2013). DynamoDB is a NoSQL database used in Amazon. It combines document and key-value data models (Sahafizadeh & Nematbakhsh, 2015).

### Security Issues in Multi-model NoSQL DBMSs

This subsection discusses the most popular types of Multi-model database Stores NoSQL Database Management Systems.

#### Cassandra

• Introduction:
  Cassandra is an open-source Multi-model database NoSQL DBMS. It was developed by Apache Software Foundation and its stable release appeared in February 2018. Cassandra was developed using Java, and is used by Facebook. Cassandra is a Multi-model database of both column stores and key-value stores (Moniruzzaman & Hossain, 2013). Its key characteristics include: a free schema, flexibility, convenience of adding or deleting fields, support for range queries, partition tolerance, and high scalability (Han et al., 2011). Cassandra has three basic components; data, centers, and clusters nodes. The data in a cluster is organized into key spaces (databases), which contain tables. Tables contain rows, and rows have columns. The query language used is Cassandra Query Language (CQL) also Cassandra supports Map-reduce querying and REST querying by Third-party APIs (Dindoliwala & Morena, 2017).
• Security Analysis:
  – Authentication: authentication in Cassandra is particularly weak since passwords are encrypted using the MD5 hash. The entire authentication in Cassandra is provided between the client and the Cassandra cluster. In other words, the inter-node message exchange does not support Authentication by default. Hence, a malicious user with access to the network used by the Cassandra cluster can bypass the client-side Authentication and extract data or damage it. However, the transmission security at the data center, rack, and a cluster is provided by Cassandra via enabling SSL/TLS in its configurations (Han et al., 2011). Cassandra supports only internal Authentication mechanisms. Cassandra 3.0 Authentication is role-based and stored internally in Cassandra system tables (Dindoliwala & Morena, 2017).
  – Authorization: The entire authorization mechanism in Cassandra is provided between the client and the Cassandra cluster (Han et al., 2011). As such, a malicious user with access to the network used by the Cassandra cluster can bypass the client-side authorization and extract data or damage it. In 2015, it had been proven that Cassandra did not support authorization (Sahafizadeh & Nematbakhsh, 2015; Paterson et al., 2006). Cassandra uses the GRANT/REVOKE mechanism to manage permissions as part of the authorization mechanism. In Cassandra 2.2 and later versions, Role-based access control is available and permissions may be applied on resources such as keyspace, table, function (Dindoliwala & Morena, 2017).
  – Auditing: Cassandra does not support auditing (Tudorica & Bucur, 2011; Paterson et al., 2006). Cassandra auditing is available in Enterprise Cassandra as a log4jbased integration and a per-node basis. Filters are available for logging using a combination of the following categories—ADMIN, AUTH, ALL, DCL, DML, and QUERY (Dindoliwala & Morena, 2017).
  – Data Encryption: Data files in Cassandra are stored without encryption and the database does not have automatic data encryption, so malicious users can access the data and read it (Nayak, Poriya & Poojary, 2013; Tudorica & Bucur, 2011). Since version 3.2, Cassandra supports at-rest data encryption through Transparent Data Encryption (TDE) (Dindoliwala & Morena, 2017).
  – SQL Injection: Cassandra Query Language (CQL) has a similar syntax to SQL’s, so it is believed that it can be attacked in the same way SQL is vulnerable to attacks by SQL injection (Nayak, Poriya & Poojary, 2013; Paterson et al., 2006).
  – Denial of service attack: Cassandra performs one thread per client. As such, it is vulnerable to denial of service attacks. A malicious user creating enough fake connections can drain Cassandra’s resources. If hackers knew the IP addresses of all Cassandra servers in the cluster and created enough fake connections, they could drive it out of service (Nayak, Poriya & Poojary, 2013).
  – Inactive connections: Cassandra does not set the timeout value for inactive connections; consequently, it has a problem in managing inactive connections (Nayak, Poriya & Poojary, 2013; Paterson et al., 2006).

#### DynamoDB

• Introduction:
  DynamoDB is a Closed source NoSQL DBMS used by Amazon. Its initial release appeared in January 2012 (Grolinger et al., 2013). DynamoDB was developed using Java (Meier & Kaufmann, 2019). It is fast and flexible and supports both the document and key-value data models (Paterson et al., 2006). The query language is proprietary in DynamoDB but MapReduce and REST query are supported (Grolinger et al., 2013).
• Security Analysis:
  – Authentication: Authentication is supported by DynamoDB (Paterson et al., 2006). It is ensured by the integration of Identity and Access Management (IAM services Grolinger et al., 2013; Paterson et al., 2006).
  – Authorization: Authorization is supported by DynamoDB (Paterson et al., 2006). It allows users to create policies and operations on domains (Grolinger et al., 2013).
  – Auditing: DynamoDB integrates with Amazon Cloud Watch Service. Access information about latencies for operations, requests throughput, and the amount of data stored (Grolinger et al., 2013) are subject to auditing at the ACWS.
  – Data Encryption: DynamoDB does not support the encryption of data at rest (Grolinger et al., 2013). Communication between the client and the server uses the https protocol (Paterson et al., 2006).

## Query Language

A query language is a set of user-entered statements that define, manipulate, and retrieve data from database and information systems by sending queries. NoSQL DBMSs don’t use a standard query language, and most of their providers have developed their query languages. This brings some stumbling blocks when users want to switch from one NoSQL DBMS to another; for instance, Cassandra supports Cassandra Query Language (CQL), while MongoDB uses Mongo query language, and so on. To resolve this, a common query language that can support a variety of NoSQL database users was needed. Unstructured Query Language, or UnQL (pronounced ‘uncle’), is a joint effort of several NoSQL DBMS providers that brings a commonplace and standardized data definition, manipulation, and retrieval language to the NoSQL platform. UnQL is being developed by the creators of Couch and SQLlite. It is considered as the superset of SQL; providing a SQL-like syntax, and familiarity to the database developers and users. The model and syntax of the UnQl is appropriate for the unstructured, self-describing data formats. UnQl provides features to fetch and process complex document structures. It also provides the elasticity of the NoSQL schema-less design and the structured table format of the relational database. Data stored in JSON format as well as in document and non-relational stores can be queried using UnQl. UnQl is open for developers and academic communities for further enhancements and development (Nayak, Poriya & Poojary, 2013).

## Comparison of NoSQL databases categories

A generic introduction to NoSQL databases through the categorization of different NoSQL databases is presented in Table 1 (Strauch, Sites & Kriha, 2011). If we add the Object Store NoSQL systems to the table, it becomes as follows:

**Table 1:** Comparison of NoSQL databases categories.

NoSQL DB categories	Performance	Scalability	Flexibility	Complexity	Functionality
Document stores	High	Variable (High)	High	Low	Variable (Low)
Key-Value Stores	High	High	High	None	Variable (None)
Graph DB	Variable	Variable	High	High	Graph Theory
Column stores	High	High	Moderate	Low	Minimal
Object Store	High	Variable (High)	High	Low	Object Oriented Programming

## Summarized Table

The aforementioned exhaustive study is summarized in Table 2, which presents the result of the comparison between the mentioned DBMSs in terms of the selected determining criteria.

**Table 2:** Summarized table.

**DBMS**	**Model**	**Company**	**Open-source**	**Implementation Programing language**	**Stable Release issue date**	**Support authentication?**	**Support authorization?**	**Support auditing?**	**Support data encryption?**	**Query types**
MongoDB	Document	MongoDB Inc.	Yes	C++, C, JavaScrip	June 2018	Yes	Yes	weak	Yes, but not support data at rest encryption	Mongo Query Language, REST, MapReduce
CouchDB	Document	apache	Yes	Erlang	November 2017	Yes	Yes	weak	No, only Client/Server SSL-based	Map reduce, REST
Couchbase Server	Document	Couchbase Inc.	Yes	Erlang, C, C++	February, 2018	Yes	No	No	No	SQL++, Map reduce, REST
Redis	Key-Value	Salvatore Sanfilippo	Yes	C, C++	June ,2018	weak	No	No	No	REST by Third-party APIs
Voldemort	Key-Value	LinkedIn	Yes	Java	July, 2017	No	No	No	No	Map reduce
Oracle BerkeleyDB	Key-Value	Oracle	No	C, C++, Java	June, 2018	No	No	No	No, only data at rest	SQLite, REST
Neo4j	Graph	Neo Technology	Yes	Java	April, 2018	No	No	No	No, only Client/Server SSL-based	Cypher, Gremlin and SparQL, REST
HyperGraphDB	Graph	Kobrix Software, Inc.	Yes	Java	May, 2017	No	No	No	No	SQL like querying, REST
AllegroGraph	Graph	Franz Inc.	No	Common Lisp	October, 2017	Yes	Yes	weak	No, only Client/Server HTTPS	SparQL and Prolog, REST
HyperTable	Column-based	Zvents before	Yes	C++	March, 2016	No	No	No	No	HQL
HBase	Column-based	apache	Yes	Java	April, 2018	Yes	Yes	Yes	No, only Client/Server	Map reduce, REST
GemStone	Object Store	GemTalk	Yes	Smalltalk	June, 2018	Yes	Yes	weak	No	No querying like SQL
db4o	Object Store	Actian	Yes	Java, C#	Sep., 2011	weak	Yes	No	weak	db4o-sql
Objectivity/DB	Object Store	Objectivity Inc	No	Java, C#, C++ and Paython	June 2017	Yes	weak	No	No	OQL
Cassandra	Multi-model	apache	Yes	Java	February, 2018	Yes	Yes	weak	Yes	CQL, Map reduce, REST by Third-party APIs
DynamoDB	Multi-model	Amazon	No	Java	2012	No	No	weak	No	Map reduce REST

## NoSQL DBMS selection algorithm

In this section, we propose a smart agent algorithm which selects NoSQL DBMSs according to the user’s needs –in our case, that would be selecting the most convenient DBMS for large medical records storage. The algorithm gives a weight to each criterion, measured as (user weight/total weights). The user sets the appropriate weights based on their needs. The algorithm arranges weights in an ascending order, and returns an ordered table according to the arranged weights.

**Result:** an ordered table (output rows)

**Input:**10 variables;

• Data Model
• Company
• Open-source
• Implementation Programming Language
• Stable Release issue date
• Support Authentication
• Support Authorization
• Support Auditing
• Support Data Encryption
• Query Types

**Inputs Weight:**

• Data Model weight
• Company weight
• Open source weight
• Implementation Programming Language weight
• Stable Release issue date weight
• Support Authentication weight
• Support Authorization weight
• Support Auditing weight
• Support Data Encryption weight
• Query Types weight

**Lists:**

• arranged weight list
• selected List
• output rows

**output:** an ordered table

**if** all input! = null then

sort inputs incrementally according to their weights.

**end**

arranged weight list = arrange (Inputs Weight).

/* Extract all tuples from the table that satisfy the user’s constraints and store data in the selected list. */

selected List= Select * from table where column name equal inputs value for each given input value.

output rows =sort selected List function (the maximum value from arrange weight list, selected list)

**Algorithm: NoSQL DBMS selection algorithm**

## Results and Discussion

LDAP is a directory service and a searchable database repository that allows authorized users and services to find information related to people, computers, network devices, and applications (Lasisi & Ajagbe, 2012). LDAP directories are used mostly for reads, and its servers are simple to install and maintain. LDAP is an Open Standard Protocol and is Lightweight. Moreover, it offers common access for multiple database management systems such that MongoDB, MySQL, PostgreSQL, Oracle 9i, IBM DB2, etc.

**Apache Directory Studio** is an integrated directory tool platform intended for use with any LDAP server, but is specifically designed to be used with ApacheDS. Creating and launching a new LDAP server now takes less than 10 s (Apache Software Foundation, 2020).

**JSON Web Token (JWT)** is an open standard that characterizes a minimized and independent approach to safely move data between parties as a JSON object. (IETF JWT, 2020).

**Spring Boot** gives a good platform to Java designers to build up an independent and production-grade spring application that you can simply run (VMware, Inc, 2020).

**Logback** is expected as a replacement to the famous log4j venture, picking up where log4j leaves off.

This paper presents a set of functions, based on web services. The latter offers a set of endpoints that include authentication, authorization, auditing, and encryption of information. It will be secure to use the client created via these web services in all types of NoSQL DBMS.

This section will explain the web services identified.

**First**, web services related to the authentication process depend on authentication through the Apache directory server. In this process, the users and units they belong to are defined within the Apache server, so that the username and password are sent via secure channel. If the user credentials are valid, JSON Web Token (JWT) returns all the user’s information including their role within the organization and the units they belong to. With the returned information, NoSQL DB allows the user to log into the database after ensuring that the user is authorized. If the validation process fails, access is denied. This web service related to the authentication process not only allows for user verification, but also provides all the processes related to the user management, such as creating a new user, updating, or deleting an existing user within the organization. The returned information might be useful for databases in one way or another. It also facilitates the interaction with the authentication server. The authentication process is done by the authentication controller, which contains two main functions: the first is the generate JWT token function, which is responsible for completing the user authentication process and returning the user’s token. The second is responsible for validating the token, and ensuring that it is valid for use and its content has not been changed or that its validity has not expired.

The other user-related processes in the authentication server are performed by the user CRUD Controller. This controller contains five main functions, which can be described as follows:

• **Get all users:** which gets all information to of all users on the authentication server and returns JWT token with the essential information, such as display name, user name, organizational unit, …etc.
• **Get user by UID:** which returns specific user’s information by the UID.
• **Delete user by UID:** which deletes any user by the UID.
• **Create a new user:** which creates a new user within the organization.
• **Update User by UID:** which updates the information of any user by UID inside the authentication server.

**Figure 2:**
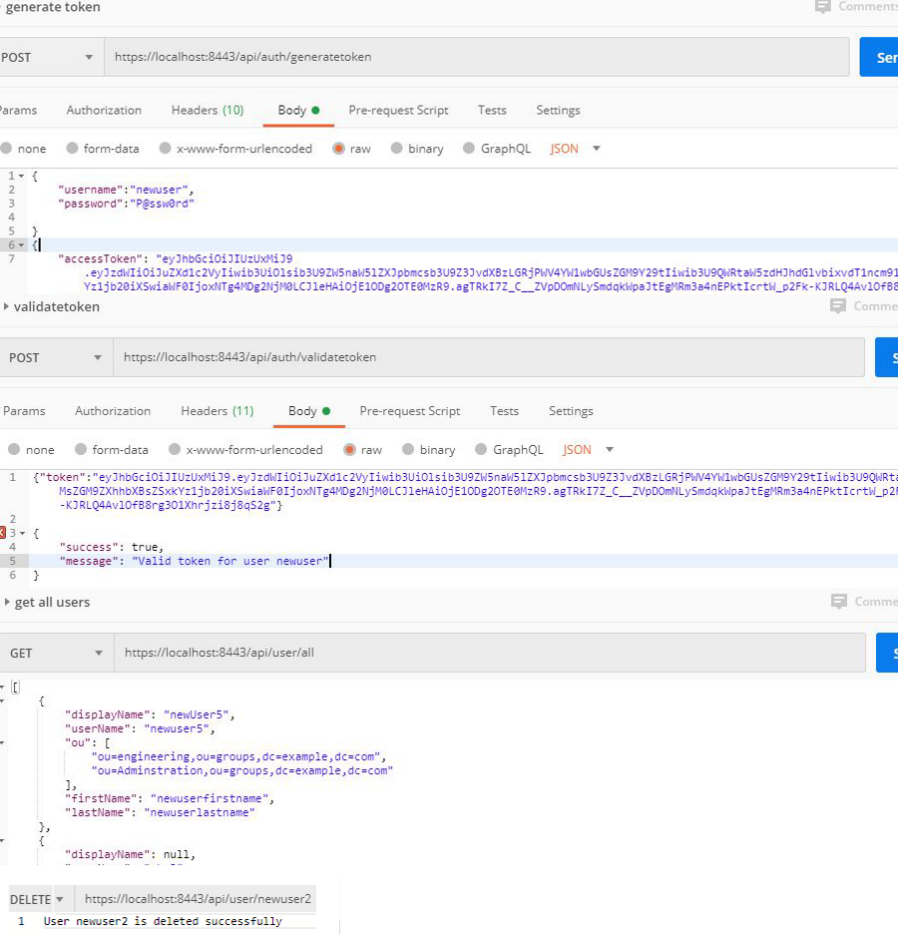
Authentication processes (a).

All processes related to the authentication processes are shown in Figs. 2. and 3.

**Figure 3:**
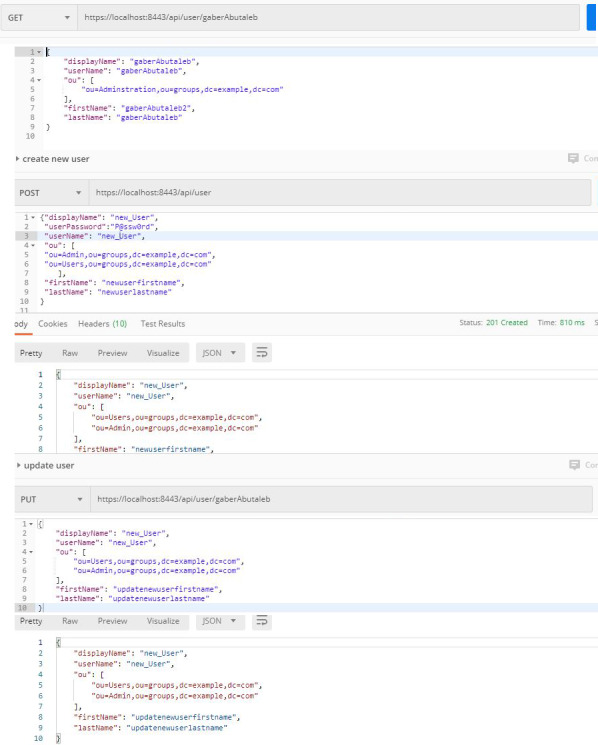
Authentication processes (b).

**Second**, the web services related to the authorization process. Most of the processes related to authorization are built within the databases. in this case, authentication is done from outside, and the user’s predefined privileges are used within the databases. But sometimes the authorized operations may be related to the roles of people within the organization, so that the databases enable users to perform the operations they are allowed to. We hereby provide a solution to authorize the usage of the role within the organization, so that they are allowed/rejected to perform their operations through their organizational units. Databases can thus allow/reject users’ operations based on their roles in the organization. The authorization process is done using an authorization controller that performs five basic functions. They can be described as follows:

• **Get all groups (organization unit):** By using this function within the NoSQL DB, the whole organization unit inside the server is returned. The returned values contain important information such as organization unit (OU) name and description.
• **Get a group by OU name:** By using this function inside the NoSQL DB, OU information is returned by the OU name.
• **Delete the group by OU name.**
• **Create a new OU:** This function enables the NoSQL DB to create a new group within the organization.
• **Update group by OU name:** This function enables updating any OU information within the apache directory server.

**Figure 4:**
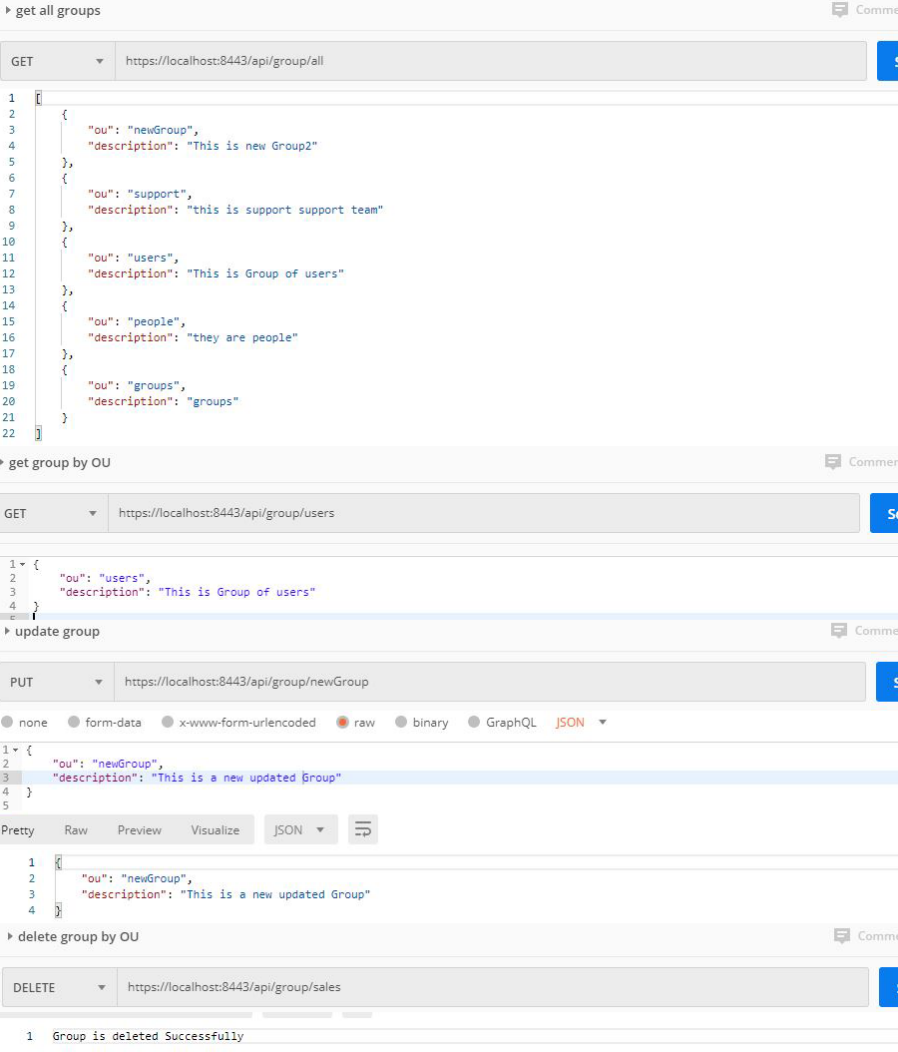
Authorization processes.

All processes related to the authorization processes are shown in Fig. 4.

**Figure 5:**
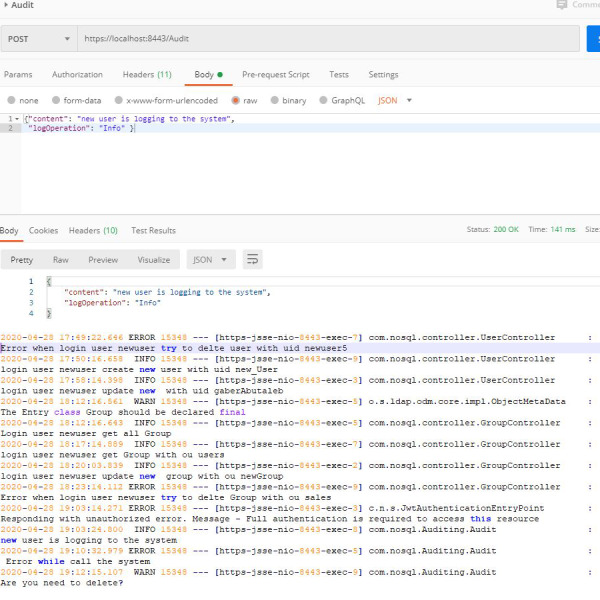
Auditing process.

**Third**, web services related to the auditing process. the auditing process is crucial for any type of database. Auditing documents all the operations that occur in the databases. We offer a set of APIs related to the auditing process, based on the Logback. The proposed solution contains the auditing controller, which contains a basic function to define the auditing process. Here is either the INFO, ERROR or WARN and the content to be stored in the auditing file. The databases will thus be able to easily record all the processes that have been performed along with their specific timestamp. Figure 5 shows one such recorded audit operations.

**Figure 6:**
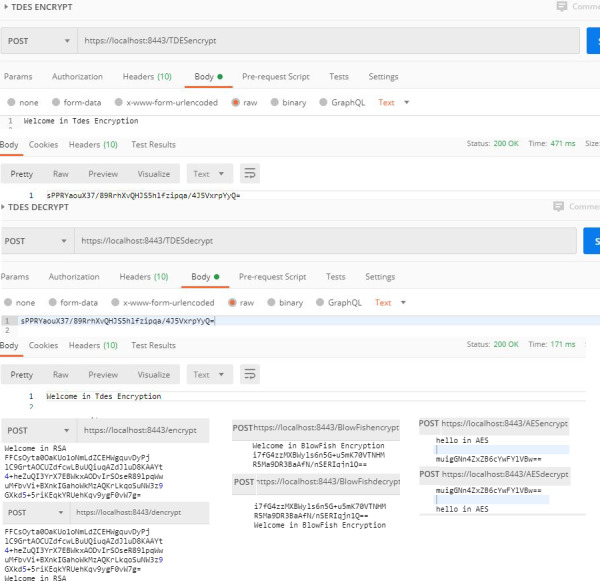
Encryption processes.

**Fourth**, web services related to the encryption process. Since securing such sensitive data as medical records is absolutely critical on DB, encryption is a basic requirement within NoSQL DB. Encryption helps to store the information safely and confidentially so that it is not being accessed by unauthorized users. This paper offers a set of web services to encrypt sensitive data, such as RSA, Triple DES, AES, and BlowFish. The web services contain four types of controllers; namely, AESController, BlowfishSsecretKey, TDESController, and RSAController. Each controller includes two functions. One is used for encryption, the other for decryption; with the possibility of storing the used keys easily and changing them if necessary. Consequently, NoSQL DB can use one of the four strongest encryption cryptosystems to encrypt sensitive data. All processes related to the encryption processes are shown in Fig. 6.

**Figure 7:**
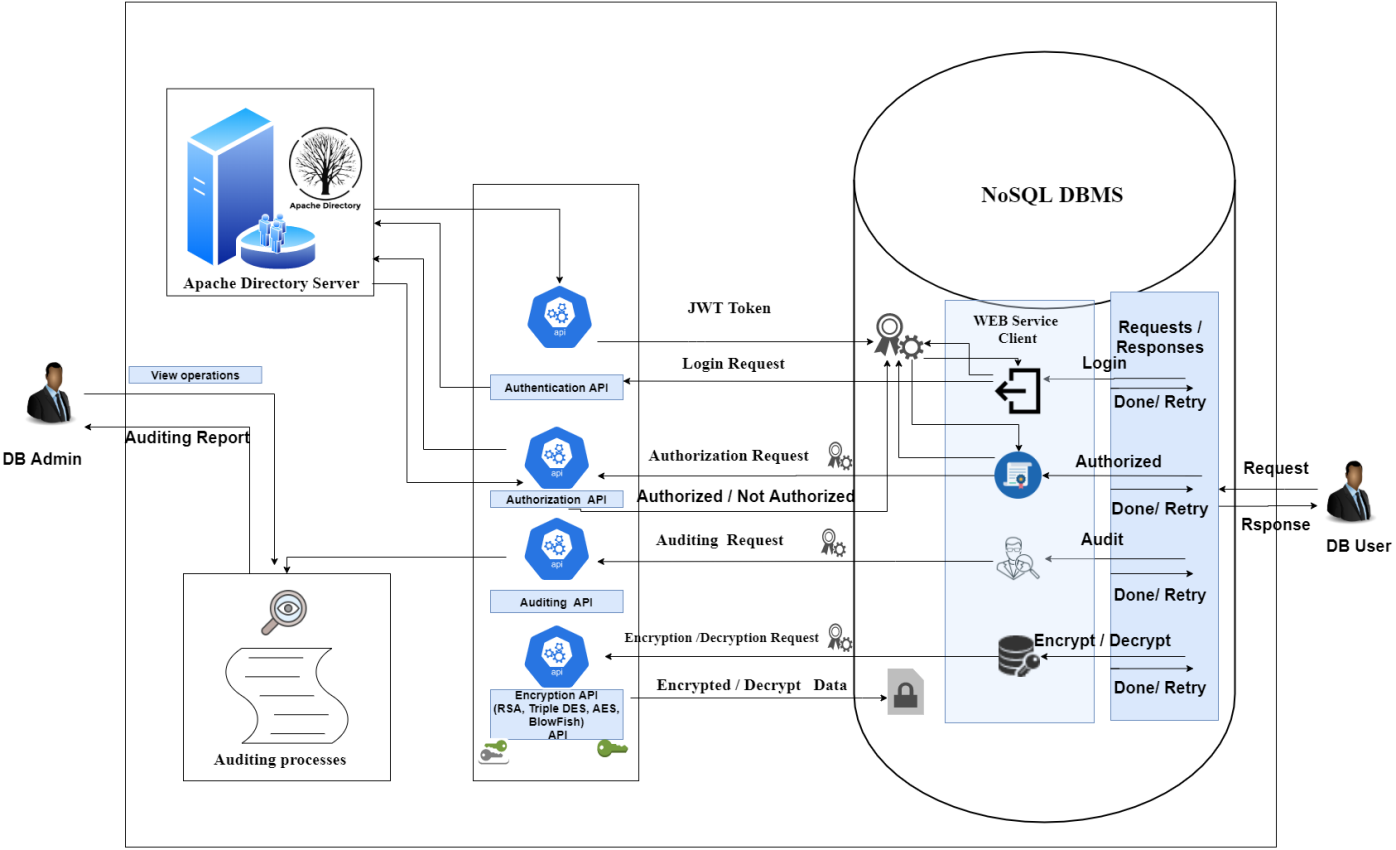
An API based security system.

The APIs described in the above paragraphs are illustrated in Fig. 7.

This figure shows the integration between the proposed system and NoSQL DBMS.

• **Login request:** In this request, the user logs in to the NoSQL DBMS. NoSQL DBMS makes a login request to the Apache directory server with the password and username sent. The Apache directory server checks the username and password and returns a JWT token in case of success; access is denied in case of failure. The JWT token is sent with all requests made to the proposed system.
• **Authorization request:** The user makes a query that requires verification of its privileges. The NoSQL DBMS checks the user’s permissions, either from the stored JWT token, or the Apache directory server. NoSQL DBMS completes or rejects the query based on the person’s authorization.
• **Auditing request:** The NoSQL DBMS uses the audit API to record all operations performed on it. The DB admin obtains the audit report to review all operations that occurred on the NoSQL DBMS.
• **Encryption/Decryption request:** The NoSQL DBMS uses the API to encrypt and decrypt all sensitive information using four different types of encryption algorithms.

It is important to mention that in the testing phase, all communications between NoSQL DB and web services will be secured with HTTPS instead of HTTP, for the data to be transmitted in a protected channel using a self-signed certificate. In the live phase, using an authentic certificate from a certificate authority is recommended.

## Contribution:

This paper presented:

1. A taxonomy of NoSQL systems categories and their DBMSs.
2. A comparative study among NoSQL DBMS, along with several criteria including security issues.
3. An algorithm to select the most convenient NoSQL DBMS for a given application. For our current purposes, this algorithm can be used to select the appropriate solution for the COVID-19 patients’, medical staff, and organizations data.
4. An API based security system. It is DBMS independent, and may be used to embed security in any NoSQL DBMS. This achieves the goal of securing COVID-19 related data.

## Conclusions

This research paves the way for the use of NoSQL databases to store and protect COVID-19 patients’ information, removing existing hurdles to their adoption. It does so by understanding the five main categories of NoSQL databases, offering a comparative study among these categories based upon a set of comparison criteria, namely Performance, Scalability, Flexibility, Complexity, Functionality, and Security issues. This comparative study reveals the strengths and weaknesses of each category. The most commonly used database management systems in each category were discussed. DBMSs were discussed from two points of view: the pertained to the information, the second the security analysis. Building on that discussion, a table was built to compare the strengths and weaknesses of each DBMS, according to their comparison criteria are Data Model, Company, Open source, Implementation Programming Language, Stable Release issue dates, Supporting Authentication, Support Authorization, Support Auditing, Support Data Encryption, and Query Types. The table provides individuals users and organizations a clear understanding of the various NoSQL DBMSs. The choice of a NoSQL DBMS will depend on its convenience to store and secure the information of COVID-19 patients. For this purpose, a new decision-support algorithm was developed, to assist in the selection process of the most secure and convenient NoSQL DBMS to store and maintain the security of the information collected on each individual patient.

The paper developed and presented a complete solution to the essential security problems common to all types of NoSQL DBMSs, thus successfully resolving their weaknesses, which previously kept them from being the favored storage solution for sensitive data. Those solutions relate to the processes of authentication, authorization, auditing, and encryption of sensitive information. Moreover, the proposed solution is sufficiently general to allow the patients’ database designer to freely choose any NoSQL DBMS to implement their design, while successfully resolving those security pitfalls.

##  Supplemental Information

10.7717/peerj-cs.297/supp-1 Supplemental Information 1 Instruction to run the code

## Additional Information and Declarations

### Data Availability

The following information was supplied regarding data availability: Code is available at GitHub: https://github.com/GaberAbutaleb/NoSQL-Authentication-API.
