# Supplementary material for: Data in the time of COVID-19: a general methodology to select and secure a NoSQL DBMS for medical data
Source: PeerJ Comput Sci. 2020 Sep 10;6:e297. doi: 10.7717/peerj-cs.297 (PMC7924412; doi:10.7717/peerj-cs.297)

1. Download the code from the below link

<https://github.com/GaberAbutaleb/NoSQL-Authentication-API>


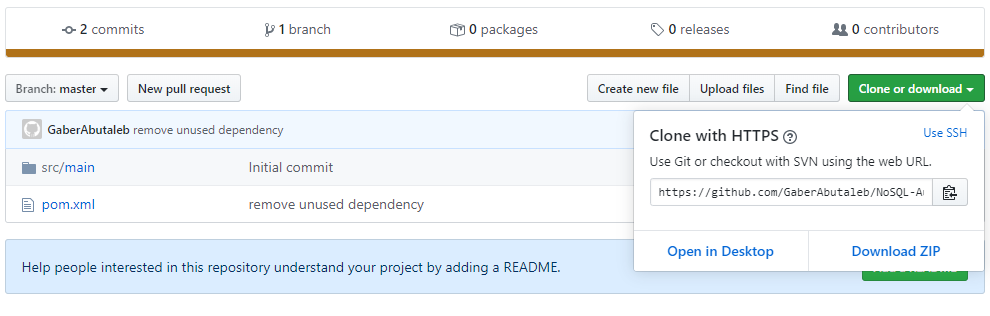


1. Download Apache Directory Studio from the below link

<https://directory.apache.org/studio/downloads.html>


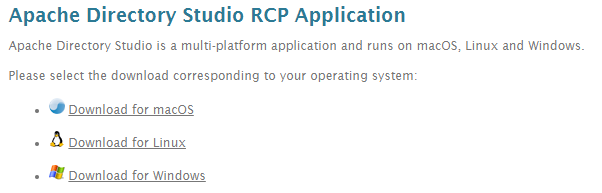


1. Setup up the Apache Directory Studio
2. Run the Apache Directory Service from the LDAP Servers tab.


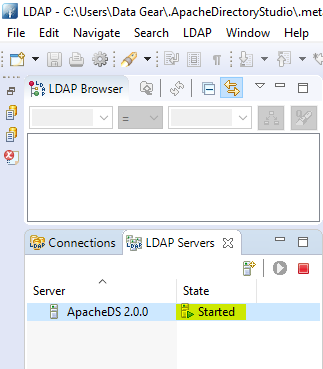


1. Create a connection to the Apache Directory Service.


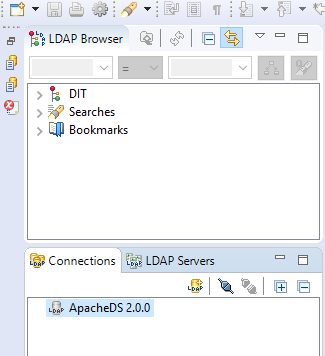


1. Expand DIT (Data information tree) to show all available domains.


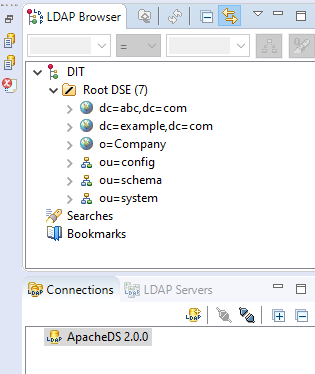


1. Import the ads.ldif to the Apache directory studio

- Right-click on the Root DES button.
- Choose the import option


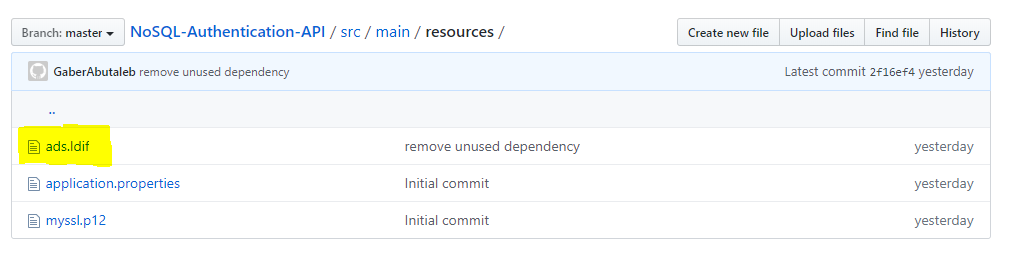


1. Now Apache directory is running on localhost under 10389 port.
2. Run the code of our application.


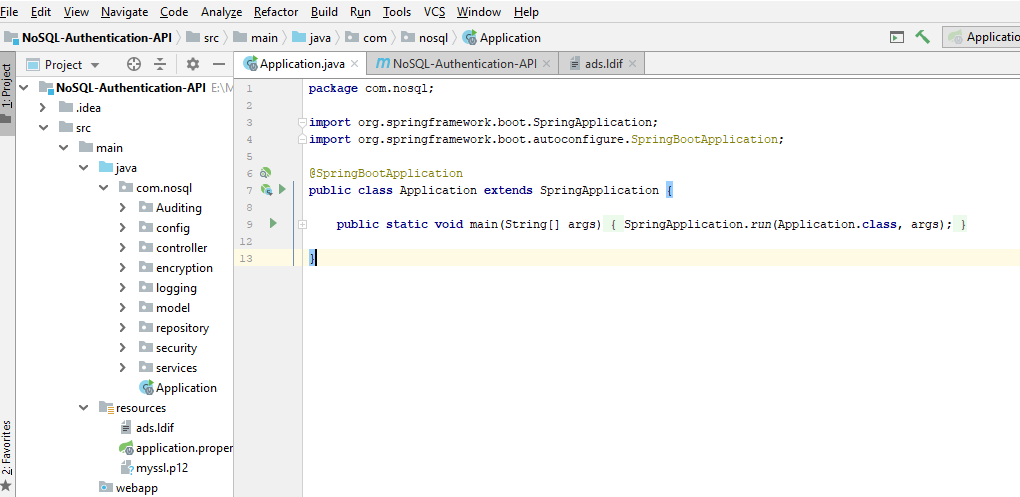


1. Now our application is ready to use by any NoSQL database Management system.


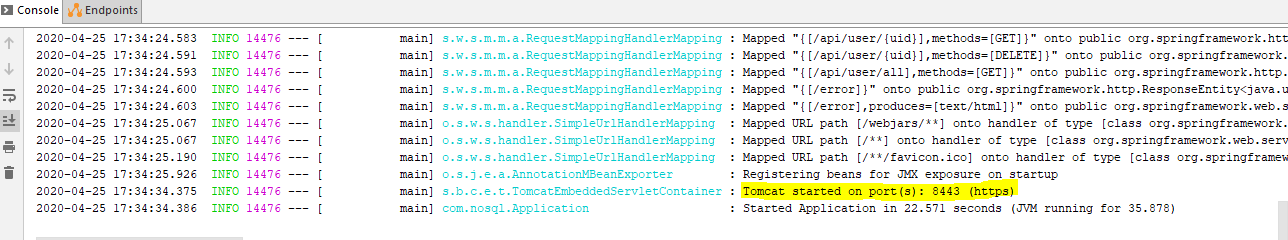

Supplement: Supplemental Information 1 [file peerj-cs-06-297-s001.docx]
